# Genome-wide miRNA expression profiling in potato (*Solanum tuberosum* L.) reveals TOR-dependent post-transcriptional gene regulatory networks in diverse metabolic pathway

**DOI:** 10.7717/peerj.10704

**Published:** 2021-01-14

**Authors:** Kexuan Deng, Huan Yin, Fangjie Xiong, Li Feng, Pan Dong, Maozhi Ren

**Affiliations:** 1 School of Life Sciences, Chongqing University, Chongqing, China; 2 Zhengzhou Research Base, State Key Laboratory of Cotton Biology, Zhengzhou University, Zhengzhou, China; 3 Institute of Urban Agriculture, Chinese Academy of Agricultural Sciences, Chengdu, China; 4 Chongqing Key Laboratory of Biology and Genetic Breeding for Tuber and Root Crops, Chongqing, China

**Keywords:** Target of rapamycin, microRNA, Integrated miRNA-mRNA analysis, Metabolism, *Solanum tuberosum* L.

## Abstract

Target of rapamycin (TOR) operates as a hub of the signal transduction that integrates nutrient and energy signaling to promote cell proliferation and growth through mediating the transcriptional and post- transcriptional regulator networks in all eukaryotic species. MicroRNAs (miRNAs) are widespread classes of small, single-stranded, non-coding endogenous RNAs and are widely found in eukaryotes, which play a vital role in regulating gene expression by degrading targeted mRNAs or translational repression at post-transcriptional level. Recent studies found that there were necessarily close connections between miRNA and TOR pathways in mammals. However, there is little information about the interplay between the miRNA and TOR in plants. Thus, the aim of this study was to identify potential TOR-miRNA-mRNA regulatory networks in TOR signaling through global mRNA and microRNA expression profiling in potato. Based on the previous high-throughput transcriptome sequencing and filtering, a total of 2,899 genes were significantly differentially expressed in potato under TOR inhibitors treatment. Pathway analysis revealed that these genes were significantly enriched in multiple metabolic processes. Similarly, in the present study, suppression of TOR resulted in 41 miRNAs up-regulated and 45 down-regulated, revealing that TOR plays a crucial role in the regulation of miRNA regulatory network. Furthermore, integrated mRNA and miRNA expression profiling uncovered that these miRNAs participated in large-scale metabolic process in the TOR signal pathway in potato, such as regulation of autophagy and ubiquitination, and biosynthesis of secondary metabolites. Overall, the results shed new insight into TOR related post-transcriptional gene regulatory networks in potato and suggesting TOR-miRNA-targeting genes relevant networks as a potential genetic resource for potato improvement.

## Introduction

MicroRNAs (miRNAs) represent an extensive class of small (usually 21–24 nucleotides length), non-coding, single-stranded, endogenous RNAs which are abundant in all eukaryotes. Since the direct cloning of first plant miRNA from *Arabidopsis* (*Arabidopsis thaliana* L.) (Park et al., 2002; Llave et al., 2002; Reinhart et al., 2002), more and more miRNAs and their putative targets were identified in different plant species, such as maize (*Zea mays* L.) (Zhang et al., 2009a), rice (*Oryza sativa* L.) (Zhang et al., 2017b; Peng et al., 2011), barley (*Hordeum vulgare* L.)  (Curaba et al., 2012), switchgrass (*Panicum virgatum* L.)  (Xie et al., 2014), wheat (*Triticum aestivum* L.)  (Han et al., 2014), sweet potato (*Ipomoea babatas* L.)  (Bian et al., 2016), rapeseed (*Brassica napus* L.)  (Jian et al., 2018), sesame (*Sesamum indicu* L.)  (Marakli, 2018) and radish (*Raphanus sativus* L.)  (Liu et al., 2018), and so on. In plants, the stem-loop secondary structure of RNA Polymerase II transcript (pri-miRNA) was cleaved by Dicer-like 1 (DCL1) protein to produce a hairpin RNA molecule (pre-miRNA), which further cleaved by DCL1 to result in a double stranded intermediate RNA (Jones-Rhoades, Bartel & Bartel, 2006 Zhu et al., 2013). The matured miRNA, which could be incorporated into the effector complex (RISC-RNA Induced Silencing Complex) can target messenger RNA to direct RNA degradation or translational inhibition (Jones-Rhoades, Bartel & Bartel, 2006; Zhu et al., 2013; Iwakawa & Tomari, 2013). MiRNAs were demonstrated to play versatile roles in plant growth, development and responding to stresses, such as temperature, drought, salt, heavy metal stresses and nutrient starvation in plants (Chen, 2009; [Bibr ref-62][Bibr ref-84]). Based on their functions in development, growth, crop yield and stress responses, they are also considered as important genetic resources in crop improvement (Liu & Chen, 2010; Tang & Chu, 2017). As one of the world’s most important staple food, potato (*Solanum tuberosum* L.) is essential to food security and human health all over the world, especially in solving the poverty (Zhang et al., 2017a). In the last decade, a fast increasing number of potato miRNAs were identified using comprehensive bioinformatic analysis of EST data, comparative genome strategy, computational prediction, and high-throughput sequencing (Zhang et al., 2013; Zhang et al., 2009b; Yang et al., 2016; Yang et al., 2010). Nevertheless, the number of the potato miRNAs (219) deposited in miRBase (http://www.mirbase.org/) was still less than that of rice (1519), *Arabidopsis* (664), cotton (*Gossypium spp* L.) (539) and maize (404). The function of some potato miRNAs were also confirmed, for example, *miR172* could induce the potato tuberization  (Martin et al., 2009), *miR396*, *miR156a*, *miR157a* and four *miR169s* were drought-induced (Yang et al., 2016; Hwang, Shin & Kwon, 2011), *miR156* could modulate potato architecture and tuberization  (Bhogale et al., 2014), *miR166* and *miR159* were responding to salinity  (Kitazumia et al., 2015), *miR482e* could enhance plant sensitivity to *Verticillium dahliae* infection  (Yang et al., 2015), *miR397-5P* was involved in the PVA infection  (Li et al., 2017), *miR164* could mediate the lateral root development  (Zhang et al., 2018), and *miR160* was associated with local defense and systemic acquired resistance against *Phytophthora infestans*  (Natarajan et al., 2018). Herein, a large number of miRNAs have yet to be discovered, and the functions of most miRNAs remain to be investigated in potato.

Target of rapamycin (TOR) was regarded as a central regulator in the signal transduction network through integrating nutrient, energy and stress related cues to coordinate cell proliferation and growth in all eukaryotic species (Xiong & Sheen, 2014; [Bibr ref-32][Bibr ref-40]). Many important downstream targets of TOR kinase have been identified in different species, such as AKT, S6K, ATG13, 4EBP1, etc (). TOR can influence gene transcription, protein translation, lipid synthesis, lysosome synthesis, autophagy and energy metabolism through these substrates (Xiong & Sheen, 2012; Laplante & Sabatini, 2012; Van Leene et al., 2019). Recent studies revealed interplays between the TOR signaling pathway and miRNAs during the occurrence and treatments of diseases in mammals  (Zhang et al., 2017b). Some miRNAs (*miR-7*, *miR-99* family, *miR-101*, *miR-122*, *miR-126*, etc.) could suppress the upstream signaling pathway of mTOR, while some miRNAs (*miR-21*, *miR-93*, *miR-96*, *miR-125b*, etc.) could activate the TOR pathway (see review; Zhang et al., 2017c). In addition, global miRNA expression profiling suggested mTOR controls many miRNAs expression in chronic rapamycin (RAP, a specific TOR inhibitor produced by *Streptomyces hygroscopicus*) treatment of cancer cells and the mouse and human cells with inactivation of TSC complex (an essential repressor of mTOR activation) (Ye et al., 2015; Totary-Jain et al., 2013; Jewell, Flores & Guan, 2015). In mammals, the miRNA biogenesis was regulated by mTORC1-Mdm2-Drosha axis in response to amino acid- and glucose- deprivation  (Ye et al., 2015). Moreover, several individual miRNAs (*miR-1*  (Sun et al., 2010), *miR-21*  (Bornachea et al., 2012), *miR-143*  (Fang et al., 2012) and *miR-125b*  (Ge, sun & Chen, 2011)) have been confirmed to be regulated by mTOR signaling, which are known to participate in some physiological functions, including cancer.

Rapamycin (RAP) was the first generation of TOR inhibitor, which could inhibit the activity of TOR only in the presence of 12-kDa FK506 binding protein (FKBP12) through forming a ternary compound RAP-FKBP12-TOR in yeast and animals  (Benjamin et al., 2011). Due to the resistance of terrestrial plants to RAP, *FKBP12* gene from *Homo sapiens* L., *Saccharomyces cerevisiae* L. or *Arabidopsis thaliana* L. was introduced into plants to generate RAP sensitive plants in the previous study (Deng et al., 2016; Sormani et al., 2007; Ren et al., 2012; Deng et al., 2017; Xiong & Sheen, 2012). In addition to RAP, the second generation of TOR inhibitors (asTORis: AZD8055, Torin1, KU63794 (KU) et al.), which could selectively and efficiently suppress TOR by specifically targeting the ATP-binding pocket of the TOR kinase domain, were also used in revealing the function of TOR in plants  (Xiong et al., 2017). In our previous study, a yeast FKBP12 gene was introduced into potato to generate RAP sensitive potato (BP12-OE line), and we found that RAP and asTORis showed synergistic effects on inhibiting potato growth and a great deal of differentially expressed genes (DEGs) were observed in potato under TOR suppression  (Deng et al., 2017). MiRNA plays an important role in post-transcriptional gene regulation in plants. Whether TOR is involved in post-transcriptional gene regulation through miRNA remains unclear in potato. Herein, we chose RAP + KU and its control DMSO to construct the potato sRNA libraries for identifying the miRNAs regulated by TOR. The integrative miRNAs and mRNA analysis was done to explicate the biological functions of TOR-miRNA-mRNA regulatory networks in potato metabolism.

## Materials and Methods

### Plant material and growth condition

In this study, the potato seedling of BP12-OE line was used for the sRNA sequencing (Deng et al., 2017). All plants were grown in Murashige & Skoog (MS) medium under 16 h light/8 h dark in growth chambers at 22 °C. Four-week-old potato seedlings growing on the MS media for 48 h with TOR inhibitors (RAP + KU) and DMSO were collected for the sRNA sequencing.

### RNA extraction, library preparation and sequencing

The extraction method and quality control of total RNA were according to Deng et al. (2017). The total amount of 3 ug RNA for each sample was used for small RNA library construction. The NEBNext Multiplex Small RNA Library Prep Set for Illumina (NEB, USA) was used to generate the sequencing libraries and the Agilent Bioanalyzer 2100 system using DNA High Sensitivity Chips was used to assess the library quality. The cBot Cluster Generation System using TruSeq SR Cluster kit v3-cBot-HS (Illumina) was used to cluster the index-coded samples. And then, the libraries were sequenced on an Illumina Hiseq 2500/2000 platform. At last, total of 50 bp single-end reads were generated.

### Data analysis

### Quality control and mapping

Raw reads of fastq format were processed through python scripts and custom perl to remove the low quality reads. The GC content, Q20 and Q30 of the raw reads were calculated. All downstream analyses were according to a range of length of clean reads, such as 18–30 nt for plant. The small RNA tags were mapped to the reference potato genome sequence (http://plants.ensembl.org/Solanum_tuberosum/Search/New?db=core) by Bowtie without mismatch to analyze their distribution and expression on the reference (Xu et al., 2011; Langmead et al., 2009).

### Bioinformatic identification of known and novel miRNAs, and their differential expression under TOR inhibitors treatment

The mapped small RNA tags were aligned to the miRNA precursor/mature miRNA of plants and animals in the miRBase20.0 database for looking for known miRNA. The potential miRNAs and their secondary structures were obtained using modified software mirdeep2  (Friedlander et al., 2012) and srna-tools-cli (http://srna-tools.cmp.uea.ac.uk/). The Custom scripts were used to analyze the known miRNA counts and their base bias either on each position or on the first position with certain length.

The rRNA, tRNA, snRNA, snoRNA, repeat and protein-coding genes were deleted from the small RNA libraries based on RepeatMasker and Rfam database. And then the remaining sequences were used for predicting the novel miRNAs with the software miREvo  (Wen et al., 2012) and mirdeep2  (Friedlander et al., 2012) through exploring the secondary structure, the minimum free energy and the Dicer cleavage site. The analysis of novel miRNA counts and their base bias was the same as that of known miRNA.

All miRNA abundances were evaluated and normalized using the tags per million reads (TPM) method through the following criteria  (Zhou et al., 2010): TPM = number of mapped miRNA reads ×10^6^/ number of clean sample reads. Differential expression analysis between the treatment and control was performed using the DESeq R package (1.8.3). The *P*-values were adjusted using *q*-value  (Storey, 2003). /log_2_fold-change/ >1 and the corrected *P*-value (*q*-value) <0.01 were set as the threshold for significantly differential expression by default.

### Prediction and annotation of target genes of miRNAs

The putative target genes of miRNAs were predicted by psRobot-tar in psRobot  (Wu et al., 2012) for plants. All the target genes were annotated in the NCBI nr database and potato genome database (http://plants.ensembl.org/Solanum_tuberosum/Search/New?db=core). For further annotating the target gene candidates of differentially expressed miRNAs, Gene Ontology (GO) enrichment and the statistical enrichment of KEGG pathways were implemented using GOseq based Wallenius non-central hyper-geometric distribution and KOBAS, respectively (Mao et al., 2005; Young et al., 2010).

### M RNA and miRNA validation by qRT-PCR

Four-week-old potato seedlings growing on the MS media were treated with TOR inhibitors (RAP + KU) and DMSO for 48 h and were collected for qRT-PCR. The mRNA validation was according to  Deng et al. (2017). The primers for qRT-PCR designed by Primer premier 5 software were listed in Table S1. To validate the expression of miRNA, 1 µg of total RNA was reverse transcribed using Mir-X™ miRNA qRT-PCR TB Green™ Kit (Takara). The RT-qPCR analysis was performed according to the manufacturer’s protocol (Mir-X™ miRNA qRT-PCR TB Green™ Kit). The miRNA-specific primers were listed in Table S1 and reverse primers was provided by kit. Potato *actin* gene was used as constitutive references (Nicot et al., 2005; Payyavula, Singh & Navarre, 2013; Liu et al., 2015). All reactions were conducted with 3 biological replicates, and the relative expression of genes was conducted using the 2^−ΔΔ*c*(*t*)^ method.

## Results

### sRNA sequencing

**Figure 1:**
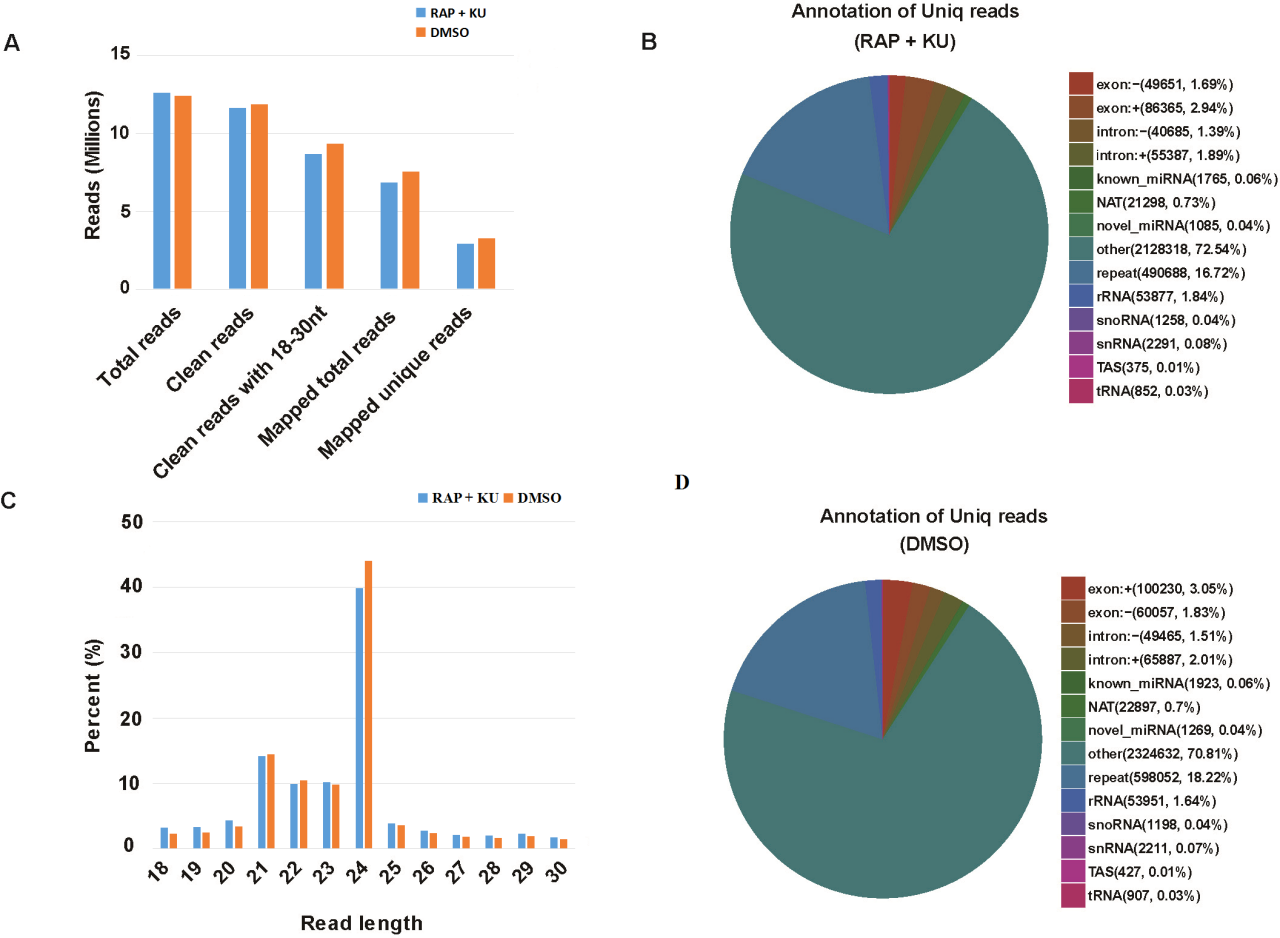
The summary of basic information for the sRNA data. (A) Statistical analysis of sequencing reads. (B) The number and proportion of different kind of sRNAs, “exon: +”: exon sense strand, “exon”: exon antisense strand, “intron: +”: intron sense strand, “intro: -”: intron antisense strand, NAT: natural antisense transcripts, repeat: repeat sequence, snoRNA: small nucleolar RNA, snRNA: small nuclear RNA, TAS: ta-si RNA. (C) The length distribution of clean sRNA.

The basic information of sRNA sequencing data from potato (BP12-OE line) with or without TOR inhibitor (RAP + KU) was shown in Fig. 1 and Table S2. The values of Q30 (bases correct recognition rate >99.9%) of the raw data were more than 97%. After removal of adapter contaminants, low quality reads, small reads and polyN, about 92.53% and 95.76% reads in total raw reads were clean reads in two groups, respectively (Fig. 1A). Finally, about 79.18% and 80.61% of the total clean reads with 18–30 nt read length could be mapped and matched to the potato genome sequence with bowtie, respectively (Langmead et al., 2009). For avoiding some sRNAs mapped to more than one annotation, the mapped clean total sRNAs were annotated according to the following priority rule: known miRNA >rRNA >tRNA >snRNA >snoRNA >repeat >NAT-siRNA >gene >novel miRNA >ta-siRNA (Fig. 1B). Most clean sRNA reads ranged from 21 to 24 nt, with 24 nt being the most abundant group of small RNAs, representing the typical length of Dicer-like protein 3 (DCL3)-derived products (Fig. 1C). The dominance of the 24 nt read length sRNA in potato is consistent with previous studies of other species, such as rice (Morin et al., 2008), cucumber (*Cucumis sativus* L.) (Mao et al., 2012), tomato (*Solanum lycopersicum* L.) (Cao et al., 2014), tobacco (*Nicotiana tabacum* L.) (Yin et al., 2015) and radish (Liu et al., 2018). The second abundant group is 21-nt miRNAs, which have the canonical size derivedfrom DCL1 processing. Most of the first nucleotide from the 5′end of the known and novel miRNAs was uridine (U), which was a bias for AGO1 (Mi et al., 2008), suggesting the important character of miRNAs was not changed under the treatment of TOR specific inhibitors (Fig. S1). In addition, in all the libraries, the total rRNA proportion was less than 60% which was used as a marker as the plant samples quality control.

### Identification of known and potential novel miRNAs in potato

In order for identifying the known miRNAs from sRNA libraries in potato, we compared the sRNA information with the known, up-dated and mature plant miRNAs deposited in the miRBase without any mismatching (Kozomara & Griffiths-Jones, 2014). Based on the blastn searches and further sequence analysis, the total of 193 known miRNA were observed and 174 known miRNA exist both in the RAP + KU and control libraries (Table S3). The read counts of the known miRNAs ranged from 0 to 24,000 in the RAP + KU and control libraries, which showed high diversity. About 17 and 22 known miRNAs were found to have more than 10,000 redundancies in the above groups, respectively (Table 1). Among these miRNAs, the miR166a-3p and miR319a-3p had the most reads.

**Table 1:** The known miRNA of more than ten thousand redundancies and novel miRNAs of more than 100 redundancies.

MiRNA	RAP+KU	DMSO	MiRNA	RAP+KU	DMSO
stu-miR319a-3p	24000	13181	novel_188	6057	11099
stu-miR166a-3p	14823	15988	novel_1	5395	9175
stu-miR166b	10331	11160	novel_16	2209	4329
stu-miR398a-3p	3832	11553	novel_17	2169	2028
stu-miR162a-3p	4850	10908	novel_18	839	1656
stu-miR396-5p	5382	5157	novel_40	665	1026
stu-miR482a-3p	2292	4388	novel_15	487	940
stu-miR6022	4287	4798	novel_30	733	764
stu-miR319b	3656	1197	novel_34	604	711
stu-miR482c	1303	2880	novel_56	280	439
stu-miR4.2e−3p	1344	2470	novel_36	574	385
stu-miR1919-5p	1679	1703	novel_38	256	322
stu-miR156a	1446	1972	novel_59	162	298
stu-miR156d-3p	1357	2244	novel_67	119	216
stu-miR6149-5p	1062	1807	novel_60	138	195
stu-miR395a	1109	1526	novel_62	128	188
stu-miR482d-3p		1325	novel_137		137
stu-miR6027		1839	novel_84		118
stu-miR482b-3p		1609	novel_68		106
stu-miR384-5p	1209		novel_58	115	
stu-miR8036-3p		1137			
stu-miR6024-3p		1060			

The available softwares miREvo (Wen et al., 2012) and mirdeep2 (Friedlander et al., 2012) were integrated to predict potential novel miRNAs. Only those with stable hairpin structures were considered, because this was an essential characteristic for identification of novel miRNAs. Additionally, the binding locations of Dicer enzymes and free energies were used to evaluate these candidate miRNAs. Ultimately, 79 novel miRNAs were discovered from two potato libraries (Table S3). The novel miRNAs displayed lower expression levels, ranging from 0 to 11,652, when compared with known miRNAs. In the RAP + KU and control group, only 17 and 19 novel miRNAs were found to have more than 100 redundancies, respectively (Table 1). In general, many novel miRNAs discoveries in potato enriched the plant miRNA dataset.

### TOR regulates potato miRNA expression

**Figure 2:**
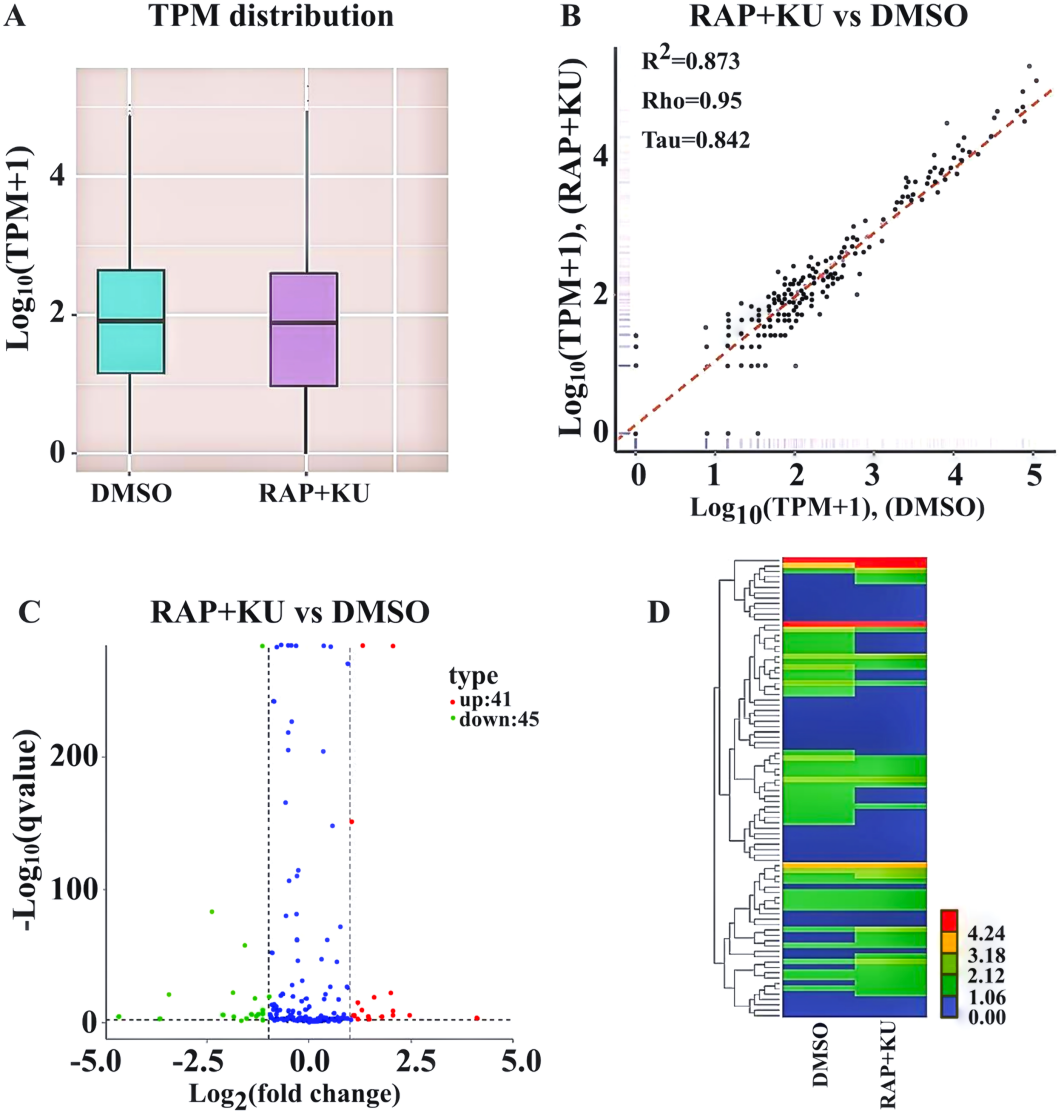
The differentially expressed miRNAs between the treatment group (RAP + KU) and control (DMSO). (A) Boxplot of the log transformed TPM expression values. (B) The correlation of miRNAs expression level between the treatment group (RAP + KU) and control (DMSO). (C) The number of differentially expressed miRNAs. (D) Hierarchical clustering of the differentially expressed miRNAs.

After obtaining the readcounts of all the miRNAs, the quantification and normalization of them were conducted by TPM (transcript per million) (Zhou et al., 2010) (Table S4). The two treatments have the similar distributions of the expression levels of all the miRNAs, and the high correlation (*R*^2^ = 0.873) of miRNA expression level showed the experimental reliability and reasonable sample selection (Figs. 2A–2B). MiRNAs with TPMs over 60 were regarded as expressing at a very high level and miRNAs with TPMs in the interval 0–1 were deemed not to be expressed at very low levels. About 86% of the total miRNAs (272) were expressed (TPM ≥ 1) and more than 146 miRNAs were highly expressed (TPM >  60) in the two treatments. Among them, one novel and 19 known miRNA were only observed in the sRNA libraries from TOR inhibition (RAP + KU), while 2 novel and 19 known miRNA were only detected in the control DMSO library, indicating TOR may completely inhibit or induce these miRNAs expression in potato.

In order to identify the differentially expressed miRNAs, we compared the expression of the known and novel miRNAs between the TOR inhibitors and control treatment samples using the DEGseq (2010) R package. Total of 86 differentially expressed miRNAs were detected in the RAP + KU vs DMSO group, including 41 up-regulated and 45 down-regulated miRNAs (Fig. 2C). To obtain the miRNA expression patterns, we performed the hierarchical clustering of all the differentially expressed miRNAs based on the log_10_ (TPMs + 1) for the two sRNA libraries (Fig. 2D). The expression levels of all the differentially expressed miRNAs were listed in Table 2 and these miRNAs (*stu-miR5303c*, *stu-miR8010*, *stu-miR8024a-3p* and *stu-miR8028-5p*) had remarkable up-regulated differences, while these miRNAs (*stu-miR3627-5p*, *stu-miR7985*, *stu-miR156f-3p* and *stu-miR8013*) had remarkable down-regulated differences. The differential expression of about 1/3 of total miRNAs suggested that TOR may play a crucial role in the regulation of miRNAs expression in potato.

### The prediction of miRNA target genes

In order to investigate the function of all the previously identified miRNAs in different biological processes, their potential target genes were predicted using psRobot_tar in psRobot for plants (Wu et al., 2012). Overall, 192 miRNAs got total 4,127 predicted target genes in 272 identified miRNAs (Table S5). Except the numbered EPlSTUG genes, all the rest (4081) numbered PGSC0003DMG genes were annotated in the website (http://plants.ensembl.org/Solanum_tuberosum/Search/New?db=core) and listed in Table S6. The number of potential targets in the potato is variable for each miRNA from 1 to 772, and most of them have multiple target genes, which are consistent with other reports (Curaba et al., 2012; Xie, Frazier & Zhang, 2011). The top three miRNAs are *stu-miR7797a* (772), *stu-miR5303h* (751) and *stu-miR5303g* (743). The results indicated the single miRNA might possess wide-ranging functions and involve in different kinds of signal pathways in potato. The products of the target genes include functional protein, transcriptional factors and enzyme, etc.

### The putative function of differentially expressed miRNAs

Some differential expression miRNAs had been reported in other species, such as miR156 and miR169 etc., (Xiong et al., 2013, Jiao et al., 2010; Meng et al., 2011; Wu et al., 2009). In addition to these miRNAs, there are a large number of miRNA functions that have not been studied. In order to understand the regulatory functions of differentially expressed miRNAs, we carried out the miRNA target prediction analysis and annotated the target genes according to the website (http://plants.ensembl.org/Solanum_tuberosum/Search/New?db=core) (Table S7). The putative miRNA target genes participated in many biological processes such as cell wall modification, the cell cycle, photosynthesis, nutrient transport, carbon and nitrogen utilization, autophagy, ubiquitination, senescence, protein and lipid metabolism, chromatin structure, hormone metabolism, signaling and stress-related processes. This result was also consistent with differentially expressed genes related to the TOR-regulated pathways reported by previous transcriptome studies (Park et al., 2002; Payyavula, Singh & Navarre, 2013; Xiong & Sheen, 2014; Caldana et al., 2013). And then Numerous GO functional and KEGG pathway enrichment of differentially expressed miRNAs were detected, suggesting TOR regulated miRNAs participated in many biological metabolic processes in potato (Tables S8A–S8B). These results suggest that miRNA may play a vital role in the regulation of metabolic processes in TOR signaling.

**Table 2:** The differentially expressed miRNAs in potato between the treatment of RAP + KU and DMSO.

**MiRNA**	**Log2 (RAP + KU)/ DMSO**	***Q* valve**	**MiRNA**	**Log2 (RAP + KU)/ DMSO**	***Q* valve**
stu-miR3627-5p	−6.01	9.80E−10	novel_36	1.03	1.08E−15
stu-miR7985	−4.69	6.26E−05	novel_99	1.04	5.16E−05
stu-miR156f-3p	−4.69	6.26E−05	stu-miR8031	1.08	1.09E−06
stu-miR8013	−4.69	6.26E−05	novel_115	1.11	4.12E−05
novel_111	−3.69	2.74E−03	stu-miR4376-3p	1.19	1.56E−15
stu-miR169a-3p	−3.69	2.74E−03	novel_53	1.19	1.34E−03
stu-miR1886 h	−3.69	2.74E−03	stu-miR172c-3p	1.19	1.34E−03
stu-miR1886i-3p	−3.69	2.74E−03	stu-miR8032a-5p	1.32	5.10E−10
stu-miR477a-3p	−3.69	2.74E−03	stu-miR319a-3p	1.32	0.00E+00
stu-miR7986	−3.69	2.74E−03	stu-miR8033-3p	1.45	7.30E−03
stu-miR7998	−3.69	2.74E−03	novel_117	1.45	1.01E−05
stu-miR8015-3p	−3.69	2.74E−03	stu-miR8020	1.45	8.78E−04
stu-miR8034	−3.69	2.74E−03	stu-miR399a-3p	1.60	1.48E−19
stu-miR8041a-5p	−3.69	2.74E−03	stu-miR8025-5p	1.78	3.42E−05
novel_154	−3.69	2.74E−03	stu-miR399j-3p	1.98	1.74E−22
stu-miR7987	−3.69	2.74E−03	stu-miR8030-5p	2.04	8.87E−07
stu-miR8023	−3.69	2.74E−03	novel_161	2.04	2.93E−09
stu-miR8050-5p	−3.69	2.74E−03	stu-miR319b	2.06	0.00E+00
stu-miR7979	−3.69	2.74E−03	stu-miR5304-5p	2.45	4.44E−06
stu-miR8004	−3.69	2.74E−03	stu-miR8008b	2.45	4.44E−06
stu-miR8045	−3.69	2.74E−03	stu-miR7995	2.45	4.44E−06
stu-miR7122-5p	−3.45	1.86E−21	stu-miR399g-3p	4.14	1.21E−03
novel_103	−2.40	8.42E−84	stu-miR8001b-3p	4.14	1.21E−03
stu-miR5303j	−2.13	9.01E−07	stu-miR8014-3p	4.14	1.21E−03
stu-miR7984c-3p	−2.13	9.01E−07	stu-miR8024a-5p	4.14	1.21E−03
stu-miR171d-5p	−1.87	2.36E−05	stu-miR7990b	4.14	1.21E−03
novel_120	−1.87	5.85E−23	stu-miR167a-3p	4.14	1.21E−03
stu-miR169f-3p	−1.87	2.36E−05	stu-miR7983-5p	4.14	1.21E−03
stu-miR8032b-3p	−1.87	2.36E−05	stu-miR166c-5p	4.14	1.21E−03
stu-miR397-3p	−1.59	6.32E−59	stu-miR8048-3p	4.14	1.21E−03
stu-miR164-5p	−1.55	4.66E−04	stu-miR8012	4.14	1.21E−03
stu-miR7991a	−1.55	4.66E−04	stu-miR5304-3p	4.14	1.21E−03
stu-miR8007a-3p	−1.55	4.66E−04	stu-miR8018	4.14	1.21E−03
stu-miR8038a-5p	−1.42	2.91E−07	stu-miR3627-3p	4.14	1.21E−03
novel_93	−1.33	3.38E−19	stu-miR6023	4.14	1.21E−03
novel_162	−1.28	3.82E−06	novel_182	4.14	1.21E−03
stu-miR7997a	−1.28	3.82E−06	stu-miR8011a-3p	4.14	1.21E−03
stu-miR172d-5p	−1.25	3.20E−07	stu-miR5303c	5.14	1.01E−05
stu-miR398a-3p	−1.14	0.00E+00	stu-miR8028-5p	5.14	1.01E−05
novel_193	−1.13	2.95E−07	stu-miR8024a-3p	5.73	9.15E−08
stu-miR399a-5p	−1.13	5.49E−03	stu-miR8010	5.73	9.15E−08
novel_116	−1.13	1.58E−10			
stu-miR5303 h	−1.13	2.39E−08			
stu-miR7980b-5p	−1.13	5.49E−03			
novel_108	−1.01	7.44E−20			

### Integrative miRNA-mRNA expression and function analysis

**Figure 3:**
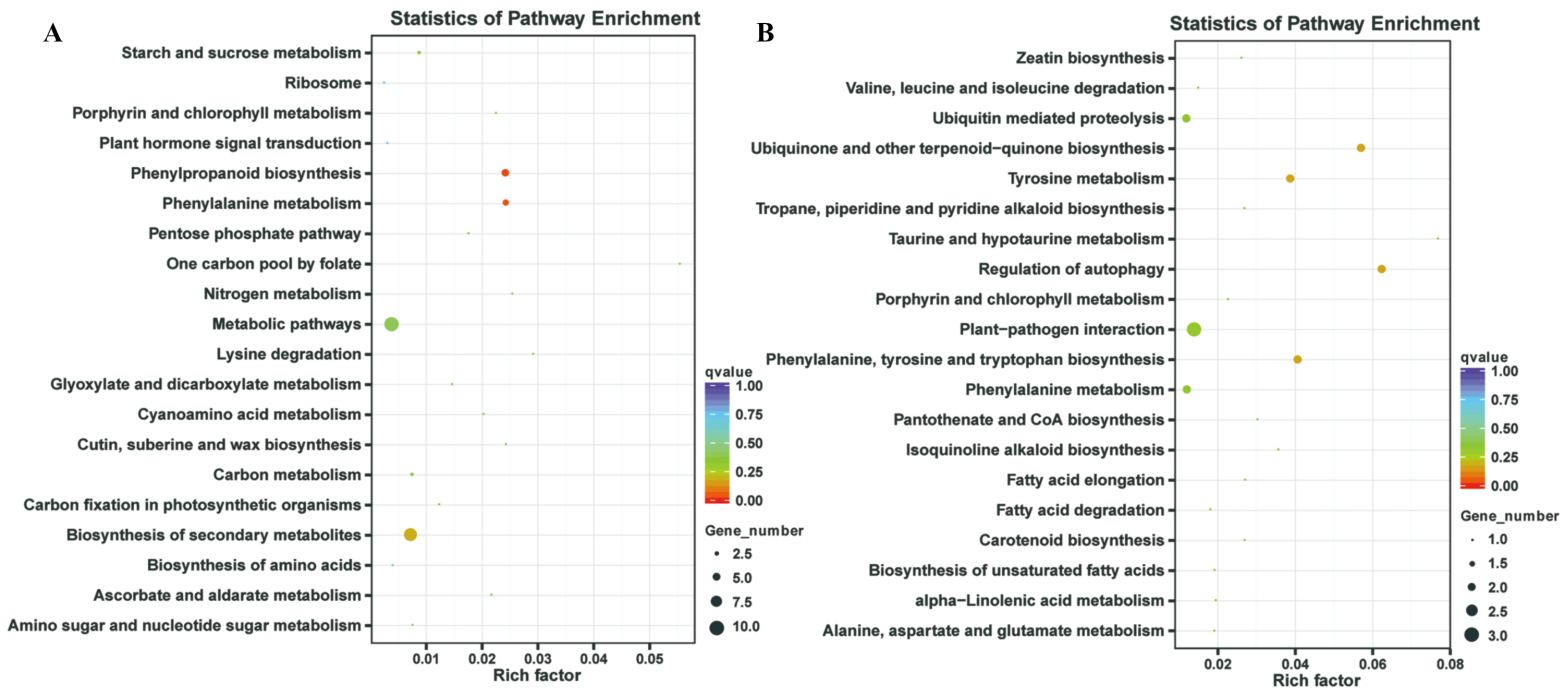
Kyoto Encyclopedia of Genes and Genomes (KEGG) enrichment analysis. (A) KEGG enrichment based on down-regulated DEGs under the TOR inhibition. (B) KEGG enrichment based on up-regulated DEGs under the TOR inhibition.

**Figure 4:**
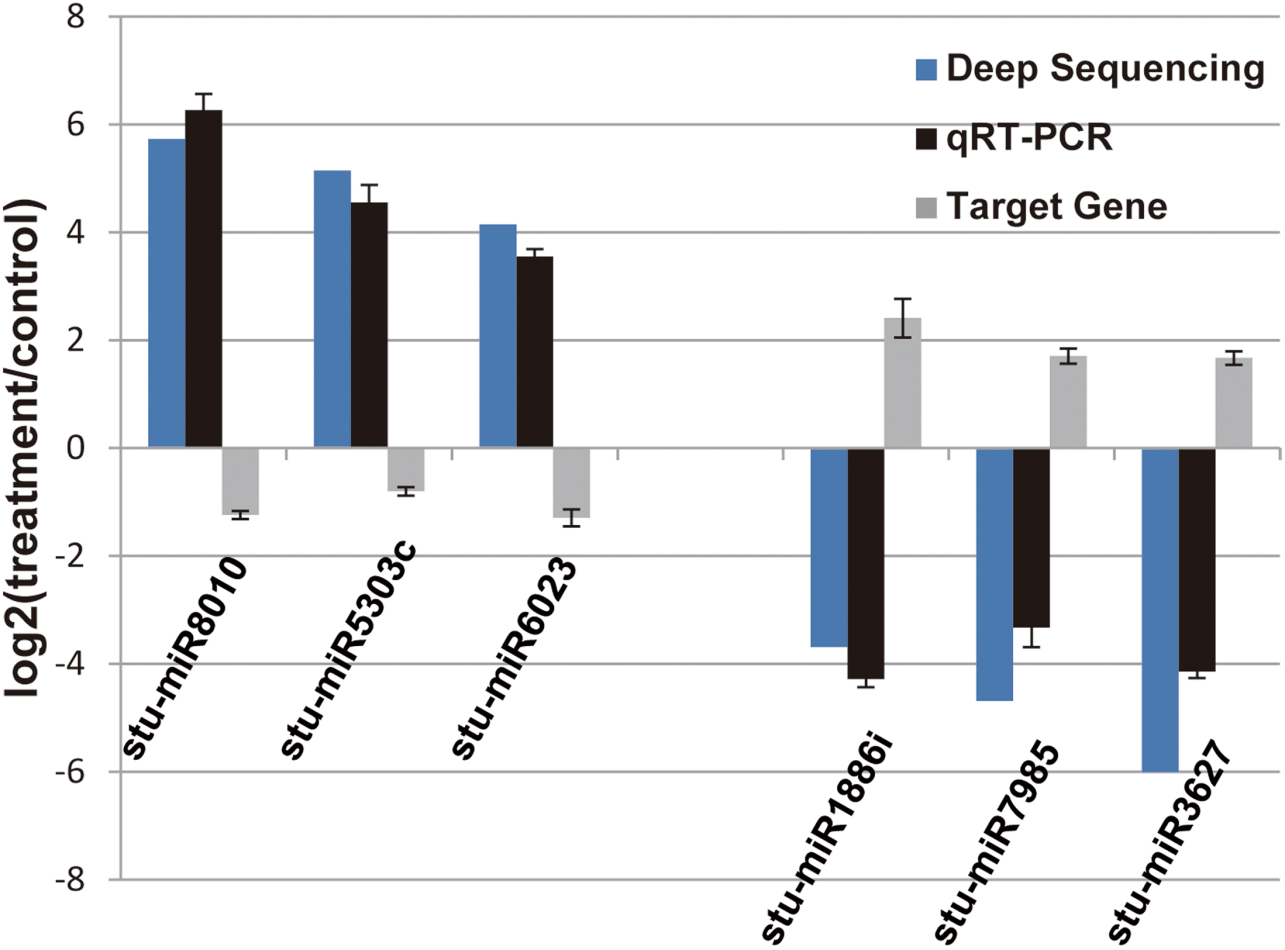
Verification of the selected miRNAs and their potential target genes by qRT-PCR. Relative expression levels of the selected six miRNAs (stu-miR8010, stu-miR5303c, stu-miR6023, stu-miR1886i, stu-miR7985 and stu-miR3627) and their potential target genes (PGSC0003DMG400026112, PGSC0003DMG400028214, PGSC0003DMG400008584, PGSC0003DMG400023703, PGSC0003DMG402018257 and PGSC0003DMG401025397) were measured by RT-qPCR. U6 and actin were used as internal control for miRNAs and mRNA RT-qPCR, respectively.

In our previous study, our focus is on the relationship between auxin, TOR and adventitious root formation (Deng et al., 2017). Our transcriptome data showed that a large number of genes associated with root development were differentially expressed in potato seedling under TOR inhibitor treatment. Loss of auxin signaling after TOR suppression results in significant down-regulation of these genes (Morin et al., 2008; Deng et al., 2017). In addition, a large number of genes related to biosynthesis and metabolism are differentially expressed. How TOR affects the expression of these genes is still unclear. To further determine the regulatory function of TOR at post-transcriptional level, we performed miRNA-mRNA reverse correlation analysis to identify reliable miRNA and their targets. In present study, thirteen up-regulated miRNAs and 14 down-regulated miRNAs were corresponding to 47 down-regulated 82 up-regulated mRNAs, respectively (Tables S9A–S9D). GO functional (Table S10A–S10B) and KEGG pathway (Fig. 3) analysis showed that the negatively correlated miRNA/mRNA interaction pairs participate in some key metabolic processes such as lipid synthesis, amino acid metabolism, nitrogen metabolism, flavonoid biosynthesis and so on. To validate the results of high-throughput sRNA sequencing, the expression patterns of 8 miRNAs were analyzed by qRT-PCR. Meanwhile, to detect the expression patterns of the targets identified by transcriptome analysis, 6 target genes were selected for qRT-PCR analysis. The results showed that all the selected miRNAs and targets shared similar expression tendency with the original results (Fig. 4).

### Differentially expressed miRNAs were involved in some anabolic and biosynthetic pathways

TOR, as a central regulator of anabolism, participates in many biosynthetic processes, such as ribosome biosynthesis, and protein translation and synthesis. In the integrative miRNA-mRNA data, DE miRNAs target genes also participate in many anabolism processes. As shown in Fig. 3 and Table 3, differentially expressed miRNAs were involved in phenylpropanoid biosynthesis, cutin, suberine and wax biosynthesis. On the other hand, TOR also negatively regulates the synthesis of some metabolites, such as glucosinolates and flavonoids. Suppression of TOR could up-regulated genes responsible glucosinolates and flavonoids synthesis (Caldana et al., 2013). It was found that the differentially expressed miRNAs target genes were also involved in the biosynthesis of flavonoids, suggesting that TOR may regulate the biosynthesis of flavonoids relying on miRNAs. Interestingly, TOR, as the core regulatory element of ribosomal biosynthesis, large number genes of ribosome was differentially expressed in transcriptomics. But only few differentially expressed miRNAs target genes were involved in ribosomal biosynthesis. These results indicated that miRNAs are only part of TOR regulation networks in transcription and post-transcriptional level.

**Table 3:** Differentially expressed miRNAs under the TOR inhibition in potato involved in catabolic process, anabolic process and biosynthetic pathways.

**MiRNA**	**Target gene_id**	**Log2FoldChange**	***P*val**	**Gene description**
**Ribosome**				
stu-miR8020	PGSC0003DMG400030153	−0.31601	1.55E−04	Ribosomal protein L18a
**Biosynthesis of amino acids**				
stu-miR7983-5p	PGSC0003DMG400022088	−0.40026	1.28E−07	Transketolase, C-terminal
**Cutin, suberine and wax biosynthesis**				
stu-miR8011a-3p	PGSC0003DMG400004844	−0.77456	4.95E−07	Glucose-methanol-choline oxidoreductase, N-terminal
**Phenylpropanoid biosynthesis**				
stu-miR4376-3p	PGSC0003DMG400020795	−2.9897	1.54E−06	Haem peroxidase, plant/fungal/bacterial
stu-miR5303c	PGSC0003DMG400003013	−0.60889	3.06E−04	Glycoside hydrolase family 3
stu-miR7983-5p	PGSC0003DMG400005872	−2.6826	2.42E−19	Plant peroxidase
stu-miR4376-3p	PGSC0003DMG400022541	−0.73675	1.21E−03	Haem peroxidase, plant/fungal/bacterial
stu-miR4376-3p	PGSC0003DMG400020801	−3.3592	4.72E−10	Peroxidases heam-ligand binding site
**Ubiquinone and other terpenoid-quinone biosynthesis**				
stu-miR7997a	PGSC0003DMG400021276	2.1196	3.17E−19	Pyridoxal phosphate-dependent transferase, major region, subdomain 1
stu-miR8045	PGSC0003DMG400017707	0.52448	3.78E−08	4-hydroxyphenylpyruvate dioxygenase
**Phenylalanine, tyrosine and tryptophan biosynthesis**				
stu-miR5303 h,stu-miR5303j	PGSC0003DMG400011282	1.4353	4.63E−03	Tryptophan synthase, beta chain, conserved site
stu-miR7997a	PGSC0003DMG400021276	2.1196	3.17E−19	Pyridoxal phosphate-dependent transferase, major region, subdomain 1
**Isoquinoline alkaloid biosynthesis**				
stu-miR7997a	PGSC0003DMG400021276	2.1196	3.17E−19	Pyridoxal phosphate-dependent transferase, major region, subdomain 1
**Pantothenate and CoA biosynthesis**				
stu-miR5303 h,stu-miR5303j,stu-miR7997a	PGSC0003DMG400013511	0.37163	1.54E−04	ATCOAA—Type II pantothenate kinase
**Tropane, piperidine and pyridine alkaloid biosynthesis**				
stu-miR7997a	PGSC0003DMG400021276	2.1196	3.17E−19	Pyridoxal phosphate-dependent transferase, major region, subdomain 1
**Fatty acid elongation**				
stu-miR8004	PGSC0003DMG400014549	0.73372	1.65E−04	Thiolase-like
**Carotenoid biosynthesis**				
stu-miR5303 h,stu-miR5303j	PGSC0003DMG402018475	1.1371	1.49E−03	Cytochrome P450
**Zeatin biosynthesis**				
stu-miR5303 h,stu-miR5303j	PGSC0003DMG400023732	1.0493	3.57E−08	UDP-glucuronosyl/UDP-glucosyltransferase
**Biosynthesis of unsaturated fatty acids**				
stu-miR8050-5p	PGSC0003DMG400020620	0.40349	8.59E−06	ACX2—Acyl-CoA dehydrogenase/oxidase C-terminal
**Lysine degradation**				
stu-miR5303c	PGSC0003DMG400001557	−0.33784	2.01E−03	Histone H3-K9 methyltransferase, plant
**Regulation of autophagy**				
stu-miR5303 h,stu-miR5303j,stu-miR7997a	PGSC0003DMG402022314	0.28852	2.09E−03	Ubiquitin-related domain
stu-miR7997a	PGSC0003DMG402012477	0.33886	3.04E−04	Autophagy protein Atg8 ubiquitin-like
**Ubiquitin mediated proteolysis**				
stu-miR5303 h,stu-miR5303j,stu-miR7997a	PGSC0003DMG400003897	0.29401	1.31E−03	Zinc finger, RING-type
stu-miR5303 h,stu-miR5303j,stu-miR7997a	PGSC0003DMG400019395	0.43331	9.66E−07	Ubiquitin-conjugating enzyme/RWD-like
**Fatty acid degradation**				
stu-miR8050-5p	PGSC0003DMG400020620	0.40349	8.59E−06	ACX2—Acyl-CoA dehydrogenase/oxidase C-terminal
**Valine, leucine and isoleucine degradation**				
stu-miR5303 h,stu-miR5303j	PGSC0003DMG400011330	0.7956	4.46E−15	BCE2—Chloramphenicol acetyltransferase-like domain

### Differentially expressed miRNAs related to some catabolic processes

Catabolic pathways is another set of metabolism that breaks down large molecules (such as lipids, polysaccharides, proteins and nucleic acids) into smaller unites (such as fatty acids, monosaccharides, amino acids and nucleotides, respectively) which are either oxidized to release energy or used in other anabolic reactions. Autophagy is a natural regulated project of the cell that disassembles unnecessary or dysfunctional components for turnover and recycling of intracellular macro molecules and whole organelles. In the mammals, many miRNAs (such as miR-145, miR-181, miR-93-5p, miR-33 and miR-21) (Zhang et al., 2017b) as the upstream regulator of TOR signaling pathway were shown to modulate disease biogenesis through inhibiting autophagy. In this study, the KEGG pathway “regulation of autophagy” was affected under the TOR suppression, and a total of three down-regulated miRNAs as the downstream of the TOR signaling pathway were targeting the autophagy-related genes (Table 3). Ubiquitination is a key posttranslational modification carried out by a set of three enzymes: ubiquitin-activating enzyme (E1), ubiquitin-conjugating enzymes (E2), and ubiquitin-protein ligase (E3). The “ubiquitin mediated proteolysis” KEGG pathway was one of the most enriched pathways in the miRNA data. A total of four differentially expressed ubiquitination-related miRNAs were observed and three were significantly down-regulated (Table 3). These observations strongly support the previous results in which TOR negatively regulated the catabolic process (autophagy and ubiquitination) in yeast, mammals and plants (Xiong & Sheen, 2014; Dong et al., 2015). In previously studies, it has showed that suppression of TOR could lead to accumulation of sugars and lipids in *Arabidopsis* (Caldana et al., 2013; Dobrenel et al., 2013). In this study, we found that many miRNA targeted genes were involved in these metabolic processes. TOR may influence the metabolic processes of sugars and lipids by regulating corresponding miRNAs. Thus, our results showed that miRNAs may act as a bridge between TOR and catabolism.

## Discussion

MiRNAs play an important role in plant growth, development and stress response. Through high-throughput sequencing technologies, many of the evolutionary conserved and novel miRNAs were detected in different species. A large number of miRNAs are still unidentified due to its low expression levels, tissue specificity or spatio-temporal expression specificity. In this study, total of 193 known miRNAs and 79 novel miRNAs were identified, largely increasing the number of miRNAs in potato. Furthermore, we found that the expression of a large number of miRNAs was affected by TOR in potato. About 86 miRNAs were differentially expressed between the libraries of TOR inhibition and the control, suggesting TOR play a crucial role in regulation the expression of miRNA. The expression levels of *stu-miR5303c*, *stu-miR8010*, *stu-miR8024a-3p* and *stu-miR166c-5p* had remarkable up-regulated differences, while that of *stu-miR3627-5p*, *novel_111*, *stu-miR169a-3p* and *stu-miR1886h*, etc., had remarkable down-regulated differences. These data suggested TOR plays a crucial role in regulation the expression of miRNA. The functions of these miRNAs have been reported in other species, such as *miR156*, *miR164* and *miR319* et al. The role of *miR156* in controlling flower development is highly conserved in rice, tomato and maize (Hong & Jackson, 2015). *MiR164* can target to *NAC* transcription factors to modulate root development and drought resistance in *Z. mays* and rice, respectively (Li et al., 2012; Fang, Xie & Xiong, 2014). Zhao et al. (2015) showed that *miR319*/*TCP4* module played a crucial role in systemic defensive response in tomato. In addition, the role of these differentially expressed miRNAs was partially revealed in potato as well, such as *miR156*, *miR172* and *miR169*. *MiR156* and *miR172* is involved in regulating developmental timing in *Arabidopsis* (Wu et al., 2009). Consistently, *miR156* and *miR172* affect not only flowering time, but also the timing of tuberization in potato. Similarly, *miR169* is conserved related with salt stress in potato and other species (Yang et al., 2016; Zhao et al., 2009; Luan et al., 2015). Indeed, it has been reported that both TOR and miRNAs are involved in many different signaling pathways to regulate plant growth and development in previous studies (Jones-Rhoades, Bartel & Bartel, 2006; Shi, Wu & Sheen, 2018). Whether TOR can regulate plant growth and development through these known miRNAs identified in this study may be a field worthy of research. In addition, many differentially expressed miRNAs identified in this study are unknown function miRNAs. Integrated mRNA and miRNA expression profiling will be helpful to predict the function of these unknown miRNAs. In this study, through the combined analysis of miRNAomics and transcriptomics, the obtained RNA-miRNA regulatory networks partially reveal the function of TOR in post-transcriptional regulation. Our data revealed more than 120 miRNA-mRNA regulatory networks involved in TOR signaling and thus provided circumstantial evidence for miRNAs involving in the role of TOR in modulating gene expression at post-transcriptional level. These data will provide valuable information for further investigation of the molecular mechanisms of miRNA involved in the TOR signaling pathway.

TOR as a core regulator of cell growth can regulate the many biological process of the organism at different levels such as: gene transcription, protein translation and synthesis and metabolic processes. A number of transcriptome analyses have showed that TOR was involved in the regulation of genes expression at transcriptional level in plants (Dong et al., 2015; Caldana et al., 2013; Song et al., 2017; Xiong et al., 2013). It had found that some transcription factors are involved in TOR signaling pathway in mammals, such as *SREBP1*, *HIF1α* and *YY1* (Laplante & Sabatini, 2012; Zoncu, Efeyan & Sabatini, 2011; Li et al., 2011; Porstmann et al., 2008; Cunningham et al., 2007). In addition, Xiong & Sheen (2014) and Xiong et al. (2013) had firstly found the transcription factors E2Fa and E2Fb which could directly activate by TOR kinase in *Arabidopsis*. However, compared with the thousands differential expression genes after TOR inhibition, transcription factors directly regulated by TOR signaling pathway are very limited. In previous studies, it has shown that miRNAs can target to signaling proteins, enzymes, transcription factors and other genes, and Cui et al. declare that miRNAs preferentially target the downstream components of the adaptors, which have potential to recruit more downstream components, such as TFs based on bioinformatics analysis (Cui et al., 2006). In this study, we found that many differentially expressed miRNAs can target to TFs, such as *miR164*, *miR156*, *miR169*, *miR172* and so on (Jiao et al., 2010; Wu et al., 2009; Zhao et al., 2009; Guo et al., 2005). In addition, we also found that some function unknown miRNAs can target to TFs based on integrated mRNA and miRNA expression profiling, such as *miR5303j*- *PGSC0003DMG400028381* (a WRKY transcription factor) module and *miR8050*- *PGSC0003DMT400021748* (a GRAS transcription factor) module. Furthermore, Cui et al. showed that variation of expression of miRNA targets is significantly lower than that of other genes in different species (Cui et al., 2007), implying that TOR-miRNA-mRNA(TF) module may be an important complement to TOR- substrate and TOR-TFs pathway in TOR-related conserved functions in eukaryotes, such as ribosome biosynthesis and lipid synthesis (Shi, Wu & Sheen, 2018). On the other hand, our data showed that differentially expressed miRNAs only participates in part of the function of TOR, suggesting that the regulation of gene transcripts by TOR signaling may rely on transcription factors at transcriptional level and other long non-coding RNA at transcriptional or post-transcriptional level. As an important cash crop, potato tubers contain abundant sugar, protein and other nutrients. TOR is a core regulator of metabolism in eukaryotes. MiRNAs also play key roles in many biological processes in animals and plants (Vaucheret, 2006; Shabalina & Koonin, 2008). How to modify these signals to regulate potato metabolism and optimize the nutritional value of potato tuber (secondary metabolites, lipid content and fatty acid composition, starch content or amino acid content) is a future course. In our current study, we predicted multiple TOR-miRNA-RNA regulatory networks for its relevance in biological metabolism. However, their roles in potato growth and development and application values in crop improvement needs further study.

## Conclusion

In conclusion, we identified 86 miRNAs that are regulated by TOR signaling in potato. Combined miRNAomics and transcriptomics analysis, more than 120 miRNA-mRNA regulatory networks were identified. These miRNAs may participate in TOR-related pathways including ribosome biosynthesis and lipid synthesis. Our results indicated a need for further experimental studies to reveal the role of TOR-related miRNAs in potato improvement.

##  Supplemental Information

10.7717/peerj.10704/supp-1 Supplemental Information 1 The first nucleotide bias for both novel and known miRNAs under the treatment of RAP +KU and DMSO

10.7717/peerj.10704/supp-2 Supplemental Information 2 Primers used in this study for mRNA and miRNA validation by qRT-PCR

10.7717/peerj.10704/supp-3 Supplemental Information 3 The basic sequencing information of sRNA libraries

10.7717/peerj.10704/supp-4 Supplemental Information 4 The known and novel miRNAs and their readcounts identified in this study

10.7717/peerj.10704/supp-5 Supplemental Information 5 The TPM of all the identified miRNA

10.7717/peerj.10704/supp-6 Supplemental Information 6 The identified miRNA and their putative targets

10.7717/peerj.10704/supp-7 Supplemental Information 7 The predicted target genes of known and novel miRNA identified in potato in this study

10.7717/peerj.10704/supp-8 Supplemental Information 8 The annotation of putative targets of differentially expressed miRNAs identified in this study

10.7717/peerj.10704/supp-9 Supplemental Information 9 Functional analysis of differentially expressed miRNAs (A) GO functional enrichments of differentially expressed miRNAs. (B) KEGG pathway enrichments of differentially expressed miRNAs.

10.7717/peerj.10704/supp-10 Supplemental Information 10 The integrative miRNAs and mRNA analysis. (A) Up-regulated miRNAs. (B) Down-regulated miRNAs. (C) Up-regulated mRNAs. (D) Down-regulated mRNAs.

10.7717/peerj.10704/supp-11 Supplemental Information 11 Functional analysis of differentially expressed mRNAs which are reverse correlation with differentially expressed miRNAs (A) The GO function analysis based on the down expressed mRNA/up expressed miRNA under the TOR inhibition with RAP + KU. (B) The GO function analysis based on the up expressed mRNA/down expressed miRNA under the TOR inhibition with RAP + KU.

## Additional Information and Declarations

### Data Availability

The following information was supplied regarding data availability: Data is available at GenBank: GSE155877.

## References

[ref-1] Benjamin D, Colombi M, Moroni C, Hall MN (2011). Rapamycin passes the torch: a new generation of mTOR inhibitors. Nature Reviews. Drug Discovery.

[ref-2] Bhogale S, Mahajan AS, Natarajan B, Rajabhoj M, Thulasiram HV, Banerjee AK (2014). MicroRNA156: a potential graft-transmissible microRNA that modulates plant architecture and tuberization in *Solanum tuberosum* ssp. andigena. Plant Physiology.

[ref-3] Bian XZE, Ma P, Jia Z, Guo X, Xie Y (2016). Identification of miRNAs in sweet potato by Solexa sequencing. Russian Journal of Plant Physiology.

[ref-4] Bornachea O, Santos M, Martinez-Cruz AB, Garcia-Escudero R, Duenas M, Costa C, Segrelles C, Lorz C, Buitrago A, Saiz-Ladera C, Agirre X, Grande T, Paradela B, Maraver A, Ariza JM, Prosper F, Serrano M, Sanchez-Cespedes M, Paramio JM (2012). EMT and induction of miR-21 mediate metastasis development in Trp53-deficient tumours. Scientific Reports.

[ref-5] Caldana C, Li Y, Leisse A, Zhang Y, Bartholomaeus L, Fernie AR, Willmitzer L, Giavalisco P (2013). Systemic analysis of inducible target of rapamycin mutants reveal a general metabolic switch controlling growth in *Arabidopsis thaliana*. The Plant Journal: for Cell and Molecular Biology.

[ref-6] Cao X, Wu Z, Jiang F, Zhou R, Yang Z (2014). Identification of chilling stress-responsive tomato microRNAs and their target genes by high-throughput sequencing and degradome analysis. BMC Genomics.

[ref-7] Chen X (2009). Small RNAs and their roles in plant development. Annual Review of Cell and Developmental Biology.

[ref-8] Cui Q, Yu Z, Purisima EO, Wang E (2006). Principles of microRNA regulation of a human cellular signaling network. Molecular Systems Biology.

[ref-9] Cui Q, Yu Z, Purisima EO, Wang E (2007). MicroRNA regulation and interspecific variation of gene expression. Trends in Genetics.

[ref-10] Cunningham JT, Rodgers JT, Arlow DH, Vazquez F, Mootha VK, Puigserver P (2007). mTOR controls mitochondrial oxidative function through a YY1-PGC-1alpha transcriptional complex. Nature.

[ref-11] Curaba J, Spriggs A, Taylor J, Li Z, Helliwell C (2012). miRNA regulation in the early development of barley seed. BMC Plant Biology.

[ref-12] Deng K, Dong P, Wang W, Feng L, Xiong F, Wang K, Zhang S, Feng S, Wang B, Zhang J, Ren M (2017). The TOR pathway is involved in adventitious root formation in *arabidopsis* and potato. Frontiers in Plant.

[ref-13] Deng K, Yu L, Zheng X, Zhang K, Wang W, Dong P, Zhang J, Ren M (2016). Target of rapamycin is a key player for auxin signaling transduction in *arabidopsis*. Frontiers in Plant Science.

[ref-14] Dobrenel T, Marchive C, Azzopardi M, Clement G, Moreau M, Sormani R, Robaglia C, Meyer C (2013). Sugar metabolism and the plant target of rapamycin kinase: a sweet operaTOR?. Frontiers in Plant Science.

[ref-15] Dong P, Xiong F, Que Y, Wang K, Yu L, Li Z, Ren M (2015). Expression profiling and functional analysis reveals that TOR is a key player in regulating photosynthesis and phytohormone signaling pathways in *Arabidopsis*. Frontiers in Plant Science.

[ref-16] Fang R, Xiao T, Fang Z, Sun Y, Li F, Gao Y, Feng Y, Li L, Wang Y, Liu X, Chen H, Liu XY, Ji H (2012). MicroRNA-143 (miR-143) regulates cancer glycolysis via targeting hexokinase 2 gene. The Journal of Biological Chemistry.

[ref-17] Fang Y, Xie K, Xiong L (2014). Conserved miR164-targeted NAC genes negatively regulate drought resistance in rice. Journal of Experimental Botany.

[ref-18] Friedlander MR, Mackowiak SD, Li N, Chen W, Rajewsky N (2012). miRDeep2 accurately identifies known and hundreds of novel microRNA genes in seven animal clades. Nucleic Acids Research.

[ref-19] Ge Y, sun Y, Chen J (2011). IGF-II is regulated by microRNA-125b in skeletal myogenesis. The Journal of Cell Biology.

[ref-20] Guo HS, Xie Q, Fei JF, Chua NH (2005). MicroRNA directs mRNA cleavage of the transcription factor NAC1 to downregulate auxin signals for *arabidopsis* lateral root development. Plant Cell.

[ref-21] Han R, Jian C, Lv J, Yan Y, Chi Q, Li Z, Wang Q, Zhang J, Liu X, Zhao H (2014). Identification and characterization of microRNAs in the flag leaf and developing seed of wheat (*Triticum aestivum* L.). BMC Genomics.

[ref-22] Hong Y, Jackson S (2015). Floral induction and flower formation–the role and potential applications of miRNAs. Plant Biotechnology Journal.

[ref-23] Hwang EW, Shin SJ, Kwon HB (2011). Identification of MicroRNAs and their putative targets that respond to drought stress in Solanum tuberosum. Journal of the Korean Society for Applied Biological Chemistry.

[ref-24] Iwakawa HO, Tomari Y (2013). Molecular insights into microRNA-mediated translational repression in plants. Molecular Cell.

[ref-25] Jewell JL, Flores F, Guan K-L (2015). Micro(RNA) Managing by mTORC1. Molecular Cell.

[ref-26] Jian H, Yang B, Zhang A, Ma J (2018). Genome-Wide Identification of MicroRNAs in response to cadmium stress in oilseed rape (Brassica napus L.) using high-throughput sequencing. International Journal of Molecular Sciences.

[ref-27] Jiao Y, Wang YD, Wang J, Yan M, Liu G, Dong G, Zeng D, Lu Z, Zhu X, Qian Q (2010). Regulation of OsSPL14 by OsmiR156 defines ideal plant architecture in rice. Nature Genetics.

[ref-28] Jones-Rhoades MW, Bartel DP, Bartel B (2006). MicroRNAS and their regulatory roles in plants. Annual Review of Plant Biology.

[ref-29] Kitazumia A, Kawahara Y, Onda TS, De Koeyer D, De los Reyes BG (2015). Implications of miR166 and miR159 induction to the basal response mechanisms of an andigena potato (Solanum tuberosum subsp, andigena) to salinity stress, predicted from network models in *Arabidopsis*. Genome.

[ref-30] Kozomara A, Griffiths-Jones S (2014). miRBase: annotating high confidence microRNAs using deep sequencing data. Nucleic Acids Research.

[ref-31] Langmead B, Trapnell C, Pop M, Salzberg SL (2009). Ultrafast and memory-efficient alignment of short DNA sequences to the human genome. Genome Bology.

[ref-32] Laplante M, Sabatini DM (2012). mTOR signaling in growth control and disease. Cell.

[ref-33] Li J, Guo G, Guo W, Guo G, Tong D, Ni Z, Sun Q, Yao Y (2012). miRNA164-directed cleavage of ZmNAC1 confers lateral root development in maize (Zea mays L.). BMC Plant Biology.

[ref-34] Li Y, Hu X, Chen J, Wang W, Xiong X, He C (2017). Integrated mRNA and microRNA transcriptome analysis reveals miRNA regulation in response to PVA in potato. Scientific Reports.

[ref-35] Li S, Ogawa W, Emi A, Hayashi K, Senga Y, Nomura K, Hara K, Yu D, Kasuga M (2011). Role of S6K1 in regulation of SREBP1c expression in the liver. Biochemical and Biophysical Research Communications.

[ref-36] Liu Q, Chen YQ (2010). A new mechanism in plant engineering: the potential roles of microRNAs in molecular breeding for crop improvement. Biotechnology Advances.

[ref-37] Liu Y, Lin-Wang K, Deng C, Warran B, Wang L, Yu B, Yang H, Wang J, Espley RV, Zhang J, Wang D, Allan AC (2015). Comparative Transcriptome Analysis of White and Purple Potato to Identify Genes Involved in Anthocyanin Biosynthesis. PLOS ONE.

[ref-38] Liu C, Liu X, Xu W, Fu W, Wang F, Gao J, Li Q, Zhang Z, Li J, Wang S (2018). Identification of miRNAs and their targets in regulating tuberous root development in radish using small RNA and degradome analyses. 3 Biotech.

[ref-39] Llave C, Xie Z, Kasschau KD, Carrington JC (2002). Cleavage of Scarecrow-like mRNA targets directed by a class of *Arabidopsis* miRNA. Science.

[ref-40] Loewith R, Hall MN (2011). Target of rapamycin (TOR) in nutrient signaling and growth control. Genetics.

[ref-41] Luan M, Xu M, Lu Y, Zhang L, Fan Y, Wang L (2015). Expression of zma-miR169 miRNAs and their target ZmNF-YA genes in response to abiotic stress in maize leaves. Gene.

[ref-42] Mao X, Cai T, Olyarchuk JG, Wei L (2005). Automated genome annotation and pathway identification using the KEGG Orthology (KO) as a controlled vocabulary. Bioinformatics (Oxford, England).

[ref-43] Mao W, Li Z, Xia X, Li Y, Yu J (2012). A combined approach of high-throughput sequencing and degradome analysis reveals tissue specific expression of microRNAs and their targets in cucumber. PLOS ONE.

[ref-44] Marakli S (2018). Identification and functional analyses of new sesame miRNAs (Sesamum indicum L.) and their targets. Molecular Biology Reports.

[ref-45] Martin A, Adam H, Diaz-Mendoza M, Zurczak M, Gonzalez-Schain ND, Suarez-Lopez P (2009). Graft-transmissible induction of potato tuberization by the microRNA miR172. Development.

[ref-46] Meng Z, Hong D, Jian-Kang Z, Fusuo Z, Wen-Xue L (2011). Involvement of miR169 in the nitrogen-starvation responses in *Arabidopsis*. New Phytologist.

[ref-47] Mi S, Cai T, Hu Y, Chen Y, Hodges E, Ni F, Wu L, Li S, Zhou H, Long C, Chen S, Hannon GJ, Qi Y (2008). Sorting of small RNAs into *Arabidopsis* argonaute complexes is directed by the 5′terminal nucleotide. Cell.

[ref-48] Morin RD, Aksay G, Dolgosheina E, Ebhardt HA, Magrini V, Mardis ER, Sahinalp SC, Unrau PJ (2008). Comparative analysis of the small RNA transcriptomes of Pinus contorta and *Oryza sativa*. Genome Research.

[ref-49] Natarajan B, Kalsi HS, Godbole P, Malankar N, Thiagarayaselvam A Siddappa, S, Thulasiram HV, Chakrabarti SK, Banerjee AK (2018). MiRNA160 is associated with local defense and systemic acquired resistance against Phytophthora infestans infection in potato. Journal of Experimental Botany.

[ref-50] Nicot N, Hausman JF, Hoffmann L, Evers D (2005). Housekeeping gene selection for real-time RT-PCR normalization in potato during biotic and abiotic stress. Journal of Experimental Botany.

[ref-51] Park W, Li J, Song R, Messing J, Chen X (2002). CARPEL FACTORY, a Dicer homolog, and HEN1, a novel protein, act in microRNA metabolism in *Arabidopsis thaliana*. Current Biology.

[ref-52] Payyavula RS, Singh RK, Navarre DA (2013). Transcription factors, sucrose, and sucrose metabolic genes interact to regulate potato phenylpropanoid metabolism. Journal of Experimental Botany.

[ref-53] Peng T, Lv Q, Zhang J, Li J, Du Y, Zhao Q (2011). Differential expression of the microRNAs in superior and inferior spikelets in rice (*Oryza sativa*). Journal of Experimental Botany.

[ref-54] Porstmann T, Santos CR, Griffiths B, Cully M, Wu M, Leevers S, Griffiths JR, Chung YL, Schulze A (2008). SREBP activity is regulated by mTORC1 and contributes to Akt-dependent cell growth. Cell Metabolism.

[ref-55] Reinhart BJ, Weinstein EG, Rhoades MW, Bartel B, Bartel D (2002). MicroRNAs in plants. Genes & Development.

[ref-56] Ren M, Venglat P, Qiu S, Feng L, Cao Y, Wang E, Xiang D, Wang J, Alexander D, Chalivendra S, Logan D, Mattoo A, Selvaraj G, Datla R (2012). Target of rapamycin signaling regulates metabolism, growth, and life span in *Arabidopsis*. Plant Cell.

[ref-57] Shabalina SA, Koonin EV (2008). Origins and evolution of eukaryotic RNA interference. Trends in Ecology & Evolution.

[ref-58] Shi L, Wu Y, Sheen J (2018). TOR signaling in plants: conservation and innovation. Development.

[ref-59] Song Y, Zhao G, Zhang X, Li L, Xiong F, Zhuo F, Zhang C, Yang Z, Datla R, Ren M, Li F (2017). The crosstalk between Target of Rapamycin (TOR) and Jasmonic Acid (JA) signaling existing in *Arabidopsis* and cotton. Scientific Reports.

[ref-60] Sormani R, Yao L, Menand B, Ennar N, Lecampion C, Meyer C, Robaglia C (2007). Saccharomyces cerevisiae FKBP12 binds *Arabidopsis thaliana* TOR and its expression in plants leads to rapamycin susceptibility. BMC Plant Biology.

[ref-61] Storey JD (2003). The positive false discovery rate: a Bayesian interpretation and the q -value. Annals of Statistics.

[ref-62] Sun G (2012). MicroRNAs and their diverse functions in plants. Plant Molecular Biology.

[ref-63] Sun Y, Ge Y, Drnevich J, Zhao Y, Band M, Chen J (2010). Mammalian target of rapamycin regulates miRNA-1 and follistatin in skeletal myogenesis. The Journal of Cell Biology.

[ref-64] Tang J, Chu C (2017). MicroRNAs in crop improvement: fine-tuners for complex traits. Nature Plants.

[ref-65] Totary-Jain H, Sanoudou D, Ben-Dov IZ, Dautriche CN, Guarnieri P, Marx SO, Tuschl T, Marks AR (2013). Reprogramming of the microRNA transcriptome mediates resistance to rapamycin. The Journal of Biological Chemistry.

[ref-66] Van Leene J, Han C, Gadeyne A, Eeckhout D, Matthijs C, Cannoot B, De Winne N, Persiau G, Van De Slijke E, Van de Cotte B, Stes E, Van Bel M, Storme V, Impens F, Gevaert K, Vandepoele K, De Smet I, De Jaeger G (2019). Capturing the phosphorylation and protein interaction landscape of the plant TOR kinase. Nature Plants.

[ref-67] Wen M, Shen Y, Shi S, Tang T (2012). miREvo: an integrative microRNA evolutionary analysis platform for next-generation sequencing experiments. BMC Bioinformatics.

[ref-68] Vaucheret H (2006). Post-transcriptional small RNA pathways in plants: mechanisms and regulations. Genes & Development.

[ref-69] Wu G, Park MY, Conway SR, Wang JW, Weigel D, Poethig RS (2009). The sequential action of miR156 and miR172 regulates developmental timing in *Arabidopsis*. Cell.

[ref-70] Wu HJ, Ma YK, Chen T, Wang M, Wang XJ (2012). PsRobot: a web-based plant small RNA meta-analysis toolbox. Nucleic Acids Research.

[ref-71] Xie F, Frazier TP, Zhang B (2011). Identification, characterization and expression analysis of MicroRNAs and their targets in the potato (Solanum tuberosum). Gene.

[ref-72] Xie F, Stewart Jr CN, Taki FA, He Q, Liu H, Zhang B (2014). High-throughput deep sequencing shows that microRNAs play important roles in switchgrass responses to drought and salinity stress. Plant Biotechnology Journal.

[ref-73] Xiong Y, McCormack M, Li L, Hall Q, Xiang C, Sheen J (2013). Glucose-TOR signalling reprograms the transcriptome and activates meristems. Nature.

[ref-74] Xiong Y, Sheen J (2012). Rapamycin and glucose-target of rapamycin (TOR) protein signaling in plants. The Journal of Biological Chemistry.

[ref-75] Xiong Y, Sheen J (2014). The role of target of rapamycin signaling networks in plant growth and metabolism. Plant Physiology.

[ref-76] Xiong F, Zhang R, Meng Z, Deng K, Que Y, Zhuo F, Feng L, Guo S, Datla R, Ren M (2017). Brassinosteriod Insensitive 2 (BIN2) acts as a downstream effector of the Target of Rapamycin (TOR) signaling pathway to regulate photoautotrophic growth in *Arabidopsis*. The New Phytologist.

[ref-77] Xu X, Pan S, Cheng S, Zhang B, Mu D, Ni P, Zhang G, Yang S, Li R, Wang J, Orjeda G, Guzman F, Torres M, Lozano R, Ponce O, Martinez D, De la Cruz G, Chakrabarti SK, Patil VU, Skryabin KG, Kuznetsov BB, Ravin NV, Kolganova TV, Beletsky AV, Mardanov AV, Di Genova A, Bolser DM, Martin DM, Li G, Yang Y, Kuang H, Hu Q, Xiong X, Bishop GJ, Sagredo B, Mejia N, Zagorski W, Gromadka R, Gawor J, Szczesny P, Huang S, Zhang Z, Liang C, He J, Li Y, He Y, Xu J, Zhang Y, Xie B, Du Y, Qu D, Bonierbale M, Ghislain M, MR Del Herrera, Giuliano G, Pietrella M, Perrotta G, Facella P, O’Brien K, Feingold SE, Barreiro LE, Massa GA, Diambra L, Whitty BR, Vaillancourt B, Lin H, Massa AN, Geoffroy M, Lundback S, DellaPenna D, Buell CR, Sharma SK, Marshall DF, Waugh R, Bryan GJ, Destefanis M, Nagy I, Milbourne D, Thomson SJ, Fiers M, Jacobs JM, Nielsen KL, Sonderkaer M, Iovene M, Torres GA, Jiang J, Veilleux RE, Bachem CW, De Boer J, Borm T, Kloosterman B, Van Eck H, Datema E, Hekkert B, Goverse A, Van Ham RC, Visser RG (2011). Genome sequence and analysis of the tuber crop potato. Nature.

[ref-78] Yang W, Liu X, Zhang J, Feng J, Li C, Chen J (2010). Prediction and validation of conservative microRNAs of Solanum tuberosum L. Molecular Biology Reports.

[ref-79] Yang L, Mu X, Liu C, Cai J, Shi K, Zhu W, Yang Q (2015). Overexpression of potato miR482e enhanced plant sensitivity to Verticillium dahliae infection. Journal of integrative plant biology.

[ref-80] Yang J, Ning Z, Zhou X, Si H, Di W (2016). Identification of four novel stu-miR169s and their target genes in Solanum tuberosum and expression profiles response to drought stress: = Entwicklungsgeschichte und Systematik der Pflanzen. Plant Systematics & Evolution.

[ref-81] Ye P, Liu Y, Chen C, Tang F, Wu Q, Wang X, Liu C-G, Liu X, Liu R, Liu Y, Zheng P (2015). An mTORC1-Mdm2-Drosha axis for miRNA biogenesis in response to glucose- and amino acid-deprivation. Molecular Cell.

[ref-82] Yin F, Qin C, Gao J, Liu M, Luo X, Zhang W, Liu H, Liao X, Shen Y, Mao L, Zhang Z, Lin H, Lubberstedt T, Pan G (2015). Genome-wide identification and analysis of drought-responsive genes and microRNAs in tobacco. International Journal of Molecular Sciences.

[ref-83] Young MD, Wakefield MJ, Smyth GK, Oshlack A (2010). Gene ontology analysis for RNA-seq: accounting for selection bias. Genome Biology.

[ref-84] Zhang B (2015). MicroRNA: a new target for improving plant tolerance to abiotic stress. Journal of Experimental Botany.

[ref-85] Zhang L, Chia JM, Kumari S, Stein JC, Liu Z, Narechania A, Maher CA, Guill K, McMullen MD, Ware D (2009a). A genome-wide characterization of microRNA genes in maize. PLOS Genetics.

[ref-86] Zhang Y, Huang B, Wang HY, Chang A, Zheng XFS (2017c). Emerging Role of MicroRNAs in mTOR Signaling. Cellular and Molecular Life Sciences.

[ref-87] Zhang W, Luo Y, Gong X, Zeng W, Li S (2009b). Computational identification of 48 potato microRNAs and their targets. Computational Biology and Chemistry.

[ref-88] Zhang R, Marshall D, Bryan GJ, Hornyik C (2013). Identification and characterization of miRNA transcriptome in potato by high-throughput sequencing. PLOS ONE.

[ref-89] Zhang H, Xu F, Wu Y, Hu H-h, Dai X-f (2017a). Progress of potato staple food research and industry development in China. Journal of Integrative Agriculture.

[ref-90] Zhang L, Yao L, Zhang N, Yang J, Zhu X, Tang X, Calderon-Urrea A, Si H (2018). Lateral root development in potato is mediated by stu-mi164 regulation of NAC transcription factor. Frontiers in Plant Science.

[ref-91] Zhang H, Zhang J, Yan J, Gou F, Mao Y, Tang G, Botella JR (2017b). Short tandem target mimic rice lines uncover functions of miRNAs in regulating important agronomic traits. Proceedings of the National Academy of Sciences of the United States of America.

[ref-92] Zhao B, Ge L, Liang R, Li W, Ruan K, Lin H, Jin Y (2009). Members of miR-169 family are induced by high salinity and transiently inhibit the NF-YA transcription factor. BMC Molecular Biology.

[ref-93] Zhao W, Li Z, Fan J, Hu C, Yang Rand Qi, X, Chen H, Zhao F, Wang S (2015). Identification of jasmonic acid-associated microRNAs and characterization of the regulatory roles of the miR319/TCP4 module under root-knot nematode stress in tomato. Journal of Experimental Botany.

[ref-94] Zhou L, Chen J, Li Z, Li X, Hu X, Huang Y, Zhao X, Liang C, Wang Y, Sun L, Shi M, Xu X, Shen F, Chen M, Han Z, Peng Z, Zhai Q, Chen J, Zhang Z, Yang R, Ye J, Guan Z, Yang H, Gui Y, Wang J, Cai Z, Zhang X (2010). Integrated profiling of microRNAs and mRNAs: microRNAs located on Xq27.3 associate with clear cell renal cell carcinoma. PLOS ONE.

[ref-95] Zhu H, Zhou Y, Castillo-Gonzalez C, Lu A, Ge C, Zhao YT, Duan L, Li Z, Axtell MJ, Wang XJ, Zhang X (2013). Bidirectional processing of pri-miRNAs with branched terminal loops by *Arabidopsis* Dicer-like1. Nature structural & Molecular Biology.

[ref-96] Zoncu R, Efeyan A, Sabatini DM (2011). mTOR: from growth signal integration to cancer, diabetes and ageing. Nature reviews. Molecular Cell Biology.

