# Supplementary figures and images for: Genome-wide miRNA expression profiling in potato (Solanum tuberosum L.) reveals TOR-dependent post-transcriptional gene regulatory networks in diverse metabolic pathway
Source: PeerJ. 2021 Jan 14;9:e10704. doi: 10.7717/peerj.10704 (PMC7811781; doi:10.7717/peerj.10704)

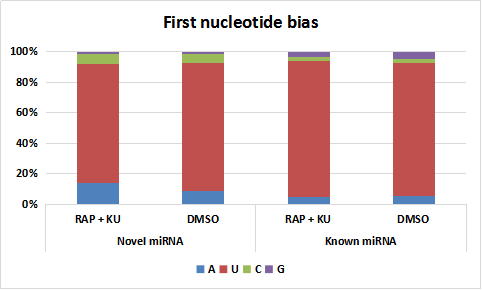

Supplement: Supplemental Information 1 [file peerj-09-10704-s001.png]
