# Supplementary material for: Genome-wide miRNA expression profiling in potato (Solanum tuberosum L.) reveals TOR-dependent post-transcriptional gene regulatory networks in diverse metabolic pathway
Source: PeerJ. 2021 Jan 14;9:e10704. doi: 10.7717/peerj.10704 (PMC7811781; doi:10.7717/peerj.10704)
Supplement: Supplemental Information 3 [file peerj-09-10704-s003.doc]

**Table S2 The basic sequencing information of sRNA libraries**

| Sample | Reads | Bases | Error rate | Q20 | Q30 | GC content |
| --- | --- | --- | --- | --- | --- | --- |
| RAP+KU | 12598581 | 0.630G | 0.01% | 98.75% | 97.60% | 48.83% |
| DMSO | 12408016 | 0.620G | 0.01% | 98.77% | 97.63% | 48.29% |
